# Flavokawain A induces deNEDDylation and Skp2 degradation leading to inhibition of tumorigenesis and cancer progression in the TRAMP transgenic mouse model

**DOI:** 10.18632/oncotarget.6166

**Published:** 2015-10-19

**Authors:** Xuesen Li, Noriko N. Yokoyama, Saiyang Zhang, Lina Ding, Hong-min Liu, Michael B. Lilly, Dan Mercola, Xiaolin Zi

**Affiliations:** ^1^ Departments of Urology, University of California, Irvine, Orange, CA, USA; ^2^ Institutes for Cancer Medicine, Sichuan Medical University, Luzhou, Sichuan, China; ^3^ Co-innovation Center of Henan Province for New Drug R & D and Preclinical Safety, Zhengzhou University, Zhengzhou, Henan, China; ^4^ Division of Hematology-Oncology, Medical University of South Carolina, Charleston, SC, USA; ^5^ Pathology and Laboratory Medicine, University of California, Irvine, Orange, CA, USA; ^6^ Chao Family Comprehensive Cancer Center, University of California, Irvine, Orange, CA, USA; ^7^ Pharmacology, University of California, Irvine, Orange, CA, USA

**Keywords:** kava, NEDDylation, Skp2, prostate cancer, TRAMP

## Abstract

S phase kinase-associated protein 2 (Skp2) has been shown to be required for spontaneous tumor development that occurs in the retinoblastoma protein (pRb) deficient mice. Here we have demonstrated that flavokawain A (FKA), a novel chalcone from the kava plant, selectively inhibited the growth of pRb deficient cell lines and resulted in a proteasome-dependent and ubiquitination-mediated Skp2 degradation. Degradation of Skp2 by FKA was found to be involved in a functional Cullin1, but independent of Cdh1 expression. Further studies have demonstrated that FKA docked into the ATP binding pocket of the precursor cell-expressed developmentally down-regulated 8 (NEDD8)-activating enzyme (NAE) complex, inhibited NEDD8 conjugations to both Cullin1 and Ubc12 in PC3 cells and Ubc12 NEDDylation in an *in vitro* assay. Finally, dietary feeding of the autochthonous transgenic adenocarcinoma of the mouse prostate (TRAMP) mice with FKA inhibited the formation of high-grade prostatic intra-epithelial neoplasia lesions (HG-PIN) and prostate adenocarcinomas, reduced the tumor burden and completely abolished distant organ metastasis. Immunohistochemistry studies revealed that dietary FKA feeding resulted in marked anti-proliferative and apoptotic effects via down-regulation of Skp2 and NEDD8 and up-regulation of p27/Kip1 in the prostate of TRAMP mice. Our findings therefore provide evidence that FKA is a promising NEDDylation inhibitor for targeting Skp2 degradation in prostate cancer prevention and treatment.

## INTRODUCTION

Prostate cancer is the second leading cause of death in man in the United States and represents a major public health burden [1]. S phase kinase-associated protein 2 (Skp2) is a substrate recognition component of a SKP1-CUL1-F-box protein (SCF) E3 ubiquitin-protein ligase complex [2]. Skp2 plays a central role in cell cycle regulation, cellular senescence, apoptosis, metabolism, metastasis, and cancer stem cells by ubiquitinating a wide range of substrates, including p27, ORC1, Cdt1, KMT2A/MLL1 and others, for targeted degradation by the proteasome [2, 3]. Skp2 has been considered as a proto-oncogene and its overexpression is frequently observed in many cancers including prostate cancer [4]. Skp2 overexpression in prostate cancer is positively associated with Gleason score, tumor grade, and biochemical failure in men treated by prostatectomy [5-7]. Overexpression of Skp2 alone in mouse prostate epithelial cells induces adenocarcinomas in the mouse prostate [8]. In metastatic and castration-resistant prostate cancer, Skp2 is a key amplified gene [9]. Importantly, multiple studies using Skp2 knockout mice have demonstrated that Skp2 is required for spontaneous tumor development that occurs in the pRb, Pten and p19Arf deficient mouse models [10-13]. Since either mutated or silenced Pten and Rb are common in prostate cancer [14, 15], Skp2 represents an appealing drug target for prostate cancer prevention and therapy.

Kava (*Piper methysticum Forst*) is an ancient drink in the South Pacific Islands [16]. An observational study has linked Kava consumption to lower cancer incidences in the South Pacific Island nations despite a high portion of smokers in these populations [17]. FKA is a predominant chalcone in the kava plant, which constitutes up to 0.46% of the kava extract [18]. Recently, we and others have demonstrated that FKA exerted potent anti-cancer and anti-carcinogenic activity in a variety of cell culture systems and animal models by induction of the mitochondria mediated apoptosis in cancer cells via up-regulation of pro-apoptotic proteins Bax and death receptor-5 (DR5) and down-regulation of anti-apoptotic proteins (i.e. survivin and XIAP) [19-22]. In addition, FKA was shown to preferentially inhibit the growth of p53 mutant bladder cancer cell lines [22]. FKA selectively induces G1 arrest in p53 wild-type bladder cancer RT4 cells *via* increasing p21/Waf1 and p27/Kip1 expression and G2M arrest in p53 mutant bladder cancer cell lines (i.e. T24, UMUC3, TCCSUP, 5637, HT1376, and HT1197) through down-regulation of Myt1 and Wee1 and activation of CDK1 [22]. However, the anti-cancer mechanisms and key molecular targets of FKA's action remain largely unknown.

In this study, we have shown that FKA selectively inhibits the growth of pRb deficient cell lines *via* inducing a proteasome-dependent and ubiquitination-mediated Skp2 degradation. In addition, FKA functions as a potent cell active NEDDylation inhibitor to induce Cullin1 and Ubc12 deNEDDylation and Skp2 degradation in all tested cancer cell lines that were derived from prostate, breast, renal, liver, lung, colon and cervical cancers, melanoma and osteosarcoma regardless of their genetic background. Finally, we found that FKA significantly reduced the formation of HG-PIN and prostate adenocarcinomas and blocked tumor metastasis in the TRAMP transgenic mice. The *in vivo* mechanisms of the action of FKA are associated with antiproliferation and induction of apoptosis via down-regulation of Skp2 and NEDD8 and up-regulation of p27/Kip1.

## RESULTS

### FKA selectively inhibits the growth of Rb deficient cells and prostate cancer cells with overexpression of Skp2

**Figure 1:**
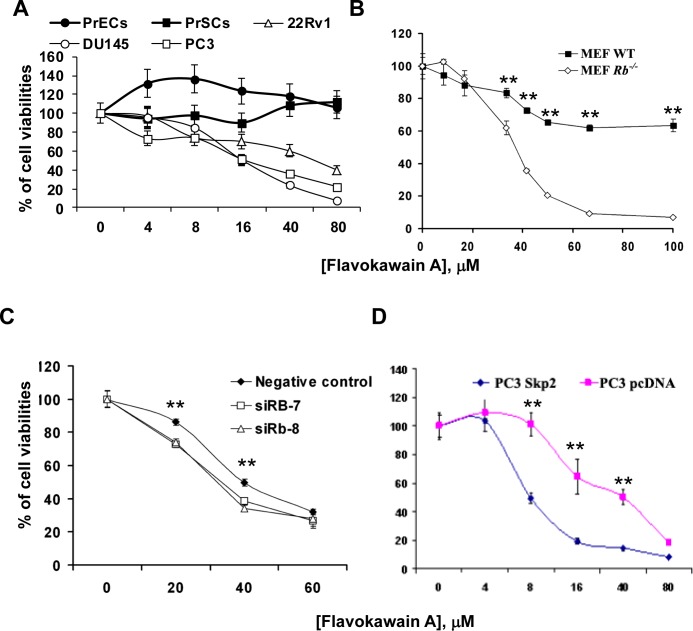
The growth inhibitory effect of FKA is associated with Rb and Skp2 expression **A**. & **B**. Prostate cancer cell lines (22Rv1, DU145 and PC3), normal prostate PrECs and PrSCs and Primary MEFs, wild-type and Rb–/–, were treated with 0.1% DMSO or indicated concentrations of FKA for 72 hours. Cell viabilities were measured by MTT assays. Points, means of four independent plates; bars, SE. **C**. Rb wild-type 22Rv1 cells were transfected with siRNA control or two Rb siRNAs (siRb-7 and -8) with different sequences to knockdown Rb expression for 48 hours. The transfected cells were also treated with 0.1% DMSO or indicated concentrations of FKA for 72 hours. Cell viabilities were measured by MTT assays as described above. **D**. PC3 cells were stably transfected with Myc-Skp2 or vector control pcDNA plasmid. Then PC3/pcDNA and PC3/Skp2 stable cell lines were treated with 0.1% DMSO or indicated concentrations of FKA for 72 hours. Cell viabilities were measured by MTT assays as described above.

We have demonstrated that FKA, a kava chalcone (Supplementary Fig. 1A), is a potent apoptosis inducer against the growth of bladder cancer cells [19], but its effect on the growth of prostate cancer cells has not been reported before. Fig. 1A shows that FKA at concentrations of up to 80 μM has no inhibitory effect on the growth of normal prostate epithelial cells (PrECs) and prostate stromal cells (PrSCs), while it inhibits the growth of prostate cancer cell lines DU145, PC3 and 22Rv1 by about 93%, 78% and 60%, respectively. DU145 cells, the only cell line among the tested cells with the Rb loss, is the most sensitive to the growth inhibitory effect of FKA. To reach the experimental rigor, we further used the Rb knockout and wild-type mouse embryonic fibroblast (MEF) pairs. At a concentration of about 64 μM, FKA treatment of Rb deficient MEFs causes about 92% growth inhibition whereas it only inhibits the growth of Rb wild-type MEFs by about 38% (Fig. 1B) (P<0.01). To confirm this phenomenon, we also treated Rb knockout mouse prostate epithelial cell line with FKA. The IC_50_ of FKA was estimated to be 30 μM in mouse epithelial prostate cells (MPEC) *Rb^−/−^* cells, compared with approximately 74 μM in MPEC wild type cells (Supplementary Fig. 1B). Furthermore, Rb knock-down by siRNAs rendered 22Rv1 cells more sensitive to the growth inhibitory effect of FKA, which also resulted in an increase in Skp2 expression and a decrease in p27 expression (Fig. 1C and Supplementary Fig. 1C). These results consistently indicate that FKA selectively inhibits the growth of Rb deficient cells.

Multiple published papers have demonstrated that Skp2 is critically required for the Rb loss induced tumorigenesis in mice [10-13]. We therefore examined whether Skp2 is a potential target responsible for FKA's growth inhibitory effect in prostate cancer cells. Fig. 1D shows that FKA is about 5 times more effective in inhibiting the growth of PC3 cells with overexpression of Skp2 (Supplementary Fig. 1D) compared to PC3 cells expressing pcDNA3.1 (IC_50s_ of FKA for PC3/Skp2 and PC3/pcDNA are about 8 and 40 μM, respectively). These data suggest that Skp2 is a potential target of FKA for inhibiting the growth of pRb deficient prostate cancer cells.

### FKA accelerates Skp2 protein degradation via a proteasome- and ubiquitination-mediated mechanism

**Figure 2:**
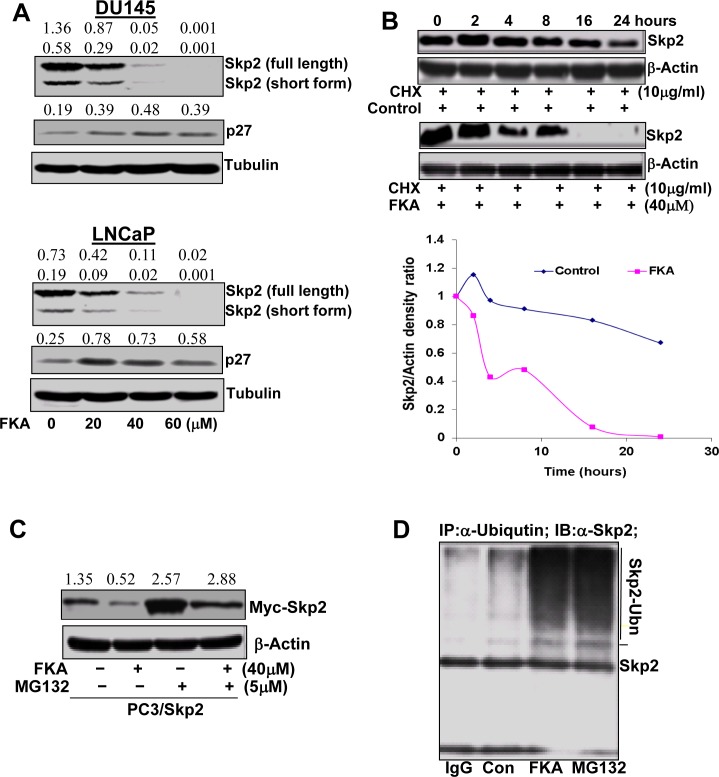
FKA accelerates Skp2 protein degradation in a proteasome and ubiquitination dependent fashion **A**. Western blotting analysis of Skp2 and its substrate (e.g. p27) expression in PC3 and LNCaP cells. β-tubulin is a loading control. A representative blotting is shown. Density ratios relative to β-tubulin were shown on the top of each Western blotting band. **B**. Skp2 protein degradation: PC3 cells were treated in the absence or presence of 40 μmol/L of FKA. After 16 h of treatment, 10μg/L of cycloheximide (CHX) was added. The protein levels of Skp2 and β-actin were analyzed by Western blotting, quantified by densitometry and plotted as a percentage of protein levels in control treatment alone for 16 hours, images and plots were representative data from three independent experiments which showed same trend. **C**. Western blotting analysis of Myc-Skp2 fusion protein expression in the PC3/Skp2 stable cell line with anti-Skp2, after FKA and MG132 treatments at indicated concentrations for 24 hours. Density ratios relative to β-tubulin were shown on the top of each Western blotting band. **D**. Skp2 ubiquitination. PC3 cells were treated with control or 40 μM FKA or 5μM MG132 for 16 hours. Ubiquitinated Skp2 was measured by immunoprecipitation of cell lysates with anti-Ubiquitin and then Western blotting analysis of immunoprecipitates by anti-Skp2.

We next examined the effect of FKA on Skp2 expression in prostate cancer cell lines. FKA treatments do not affect Skp2 expression at the transcriptional levels (data not shown), but down-regulates the protein expression of Skp2 and induces the accumulation of p27 protein, a Skp2 substrate, in prostate cancer cell lines in a dose and time manner (Fig. 2A and Supplementary Fig. 2). Two Skp2 splicing variants were detected at 46 and 48 kDa and both decreased by FKA treatment (Fig. 2A and Supplementary Fig. 2).

Fig. 2B demonstrates that Skp2 degradation is significantly accelerated in FKA-treated PC3 cells as compared to the control treatment after new protein synthesis was inhibited by cycloheximide. The protein expression levels of Skp2 at different time points after cyloheximide and FKA treatments were measured by densitometry measurement and adjusted for loading control β-actin levels (Fig. 2B). To examine whether the FKA mediated down-regulation of Skp2 protein expression is due to active proteasome–dependent degradation (a transcriptionally-independent mechanism), we have stably expressed Skp2-myc fusion protein in PC3 cells, which is driven by an exogenous CMV promoter. The PC3/Skp2 cells were then treated with 40 μM FKA for 16 hours followed by 0.1%DMSO or a proteasome inhibitor MG132. Fig. 2C shows that MG132 can rescue the effect of the FKA induced down-regulation of Skp2-myc fusion protein, suggesting a mechanism of proteasome-dependent degradation.

We also examined whether Skp2 degradation by FKA is associated with ubiquitination. Extensive Skp2 ubiquitin conjugates, as defined by the appearance of more slowly migrating forms of Skp2, were observed after FKA or a proteasome inhibitor MG132 (a positive control) treatment (Fig. 2D). Together, these results clearly indicate that FKA induces a proteasome dependent and ubiqutination mediated Skp2 degradation in PC3 cells.

### FKA induced Skp2 degradation is independent of Cdh1 expression, but associated with the functional Skp2/Cullin1 complex

**Figure 3:**
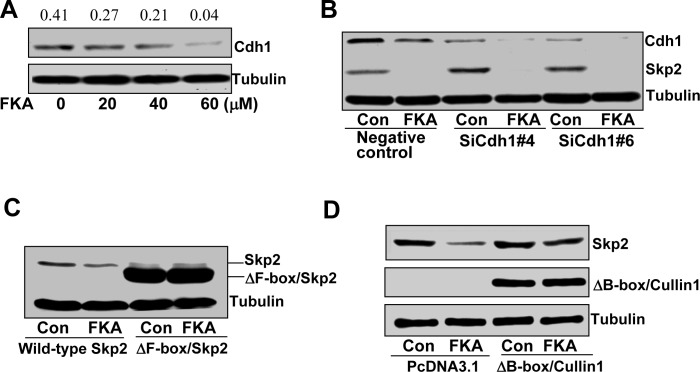
A B-box deletion-Cullin1 but not Cdh1 siRNA rescues the effect of FKA induced Skp2 degradation **A**. PC3 cells were treated with 0.1%DMSO or 20, 40 and 60 μM FKA for 24 hours. Cdh1 expression was analyzed by Western blotting. Density ratios relative to β-tubulin were shown on the top of each Western blotting band. **B**., **C**. and **D**. PC3 cells were transfected with vector control, siRNA controls, or Cdh1 siRNAs, or F-box deletion Skp2 (V5-del-F-box-Skp2) or B-box deletion Cullin1[flag-Dominant negative-Cullin-1, which only expression the N terminal region (1-456)]. After 24 h transfections, cells were treated with 0.1%DMSO or 40 μM FKA for 16 hours. SKp2 expression was examined by Western blotting.

Skp2 ubiquitination and degradation can be mediated by either (1) the Cdh1/APC E3 complex [23] or (2) through an autocatalytic mechanism involving a Skp2-bound Cullin1-based core ubiquitin ligase [24]. As a first step towards defining the mechanism of FKA mediated Skp2 degradation, we examined whether Cdh1 expression is required. FKA results in a decrease in Cdh1 expression (Fig. 3A). However, suppression of Cdh1 by siRNAs increases Skp2 protein levels, and does not reverse the FKA mediated Skp2 degradation (Fig. 3B). These results suggest that Cdh1 expression is not required for FKA mediated Skp2 degradation.

The autocatalytic ubiquintination and degradation of Skp2 requires both the binding of Skp2 to Cullin1 and the ability of the Cullin1 to recruit Cdc34 and Roc1/Rbx through its B-box motifs [24]. We therefore expressed an F-box deleted Skp2 and a B-box deleted Cullin1, respectively, in PC3 cells, and then examined the effect of FKA on Skp2 or ΔFbox/Skp2 protein expression. Fig. 3C shows that FKA treatment does not stimulate the degradation of ΔFbox/Skp2 protein that cannot bind to Cullin1. In addition, the B-box deleted Cullin1 reverses the effect of FKA on Skp2 degradation (Fig. 3D). These results clearly indicate that Cullin1 is involved in FKA-mediated Skp2 degradation.

### FKA inhibits Cullin1 and Ubc12 NEDDylation in prostate cancer cells

**Figure 4:**
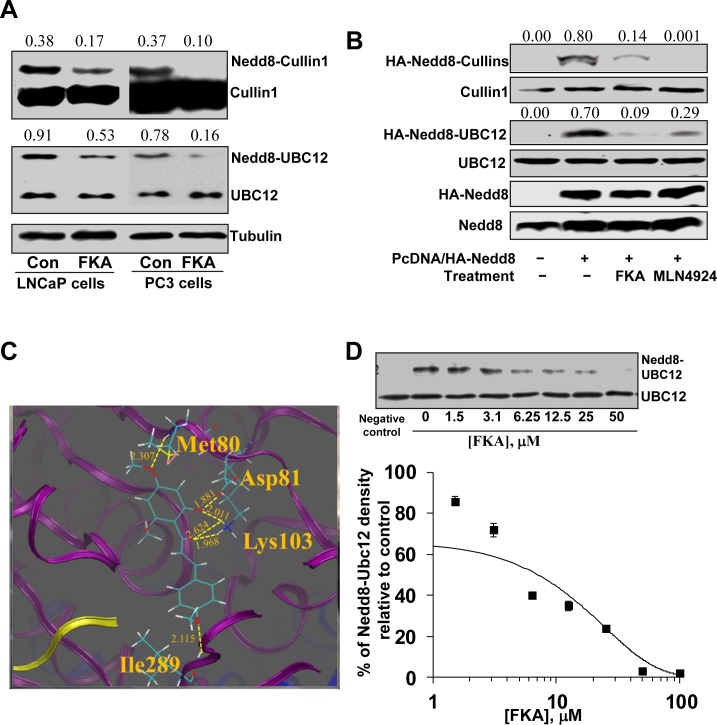
The effect of FKA on NEDDylation **A**. PC3 cells and LNCaP cells were treated with 40 μM FKA for 16 hours. Western blot analysis shows the bands detected by anti-Cullin-1 antibody, the upper bands are Nedd8 modified Cullin-1. Density ratios relative to β-tubulin were shown on the top of each Western blotting band. **B**. PC3 cells were transfected with Myc tagged NEDD8. 24 hours after transfection, cells were treated with vehicle control or 40 μM FKA or 1μM NAE inhibitor MLN4924 for 16 hours. Neddylation and expression of Cullin1, Ubc12 and Nedd8 were examined by Western blotting analysis with anti-HA, -Cullin1, -Ubc12 and -NEDD8 antibodies, respectively. Density ratios relative to β-tubulin were shown on the top of each Western blotting band. **C**. Low-energy binding conformation of FKA bound to NAE heterodimer generated by Surflex-Dock method. Proteins APPB1 (blue), UBA3 (purple) and NEDD8 (yellow) are displayed in the ribbon form. Small molecules are depicted as a line model showing carbon (light green), hydrogen (white), oxygen (red) atoms. H-bonds are indicated as dashed yellow lines. The residues which form H-bonding with the small molecule are also depicted as a line model. **D**. *In vitro* Ubc12 NEDDylation assay was carried out using the NEDD8 Conjugation Initiation Kit from Boston BioChem (Cambridge, MA) [31]. NEDD8 was added into the reaction buffer containing NEDDylation enzymes (His-APP-BP1, His-Uba3, and Ubc12) with indicated concentrations of FKA. Non-reducing western blot were performed with anti-UbcH12 antibody to detect both Ubc12 bands and Nedd8 conjugated Ubc12 bands. Densitometry analysis of the Western blot showing FKA mediated percentage inhibition of Ubc12–NEDD8 with an estimated IC_50_ of approximately 5 μM.

Cullin1 NEDDylation is processed by the NAE transfer of NEDD8 to E2 Ubc12 and then from Ubc12 to Cullin1 [25]. Fig. 4A shows that FKA treatment of LNCaP and PC3 cells for 24 hours significantly decreased the endogenously NEDDylation of Cullin1 and Ubc12. Further experiment also showed that FKA and a known NAE inhibitor, MLN4924, treatments markedly inhibits the induced NEDDylation of both Cullin1 and Ubc12 by transfection of NEDD8 into PC3 cells, as evident by the appearance of less slowly migrating forms of Cullin1 and Ubc12 (Fig. 4B).

### FKA docks into the ATP binding site of the APPBP – UBA3–NEDD8 protein complex and inhibits Ubc12 NEDDylation *in vitro*

The X-ray crystal structure of the quaternary APPBP1–UBA3–NEDD8–ATP complex (PDB ID: 1R4N) [26] was used as the initial structure for the computational study. The flexible ligand was docked to a rigid receptor and assigned a score reflecting the quality of the complex according to Surflex-dock method (Sybyl-x2.0 program). Currently, the favorable binding pose according to the highest-scoring of FKA in the APPBP1–UBA3–NEDD8 complex is shown in Fig. 4C.

In the current docking simulation, the result shows that there are four groups of FKA that can form H-bonds with the surrounding residues of amino acids. In the upper ring of FKA, the 4′-OCH_3_ group forms a H-bond with H-N (Met80) (2.307 Å) while 6′-OH group forms a H-bond with Asp81 (1.881 Å) and Lys103 (2.011 Å), respectively. The carbonyl group of FKA forms two H-bonds (2.624 Å and 1.968 Å) with the NH3 group of Lys103, respectively. In addition, the 4-OCH_3_ group in the bottom ring of FKA forms a H-bond (2.115 Å) with NH group of Ile289.

In an *in vitro* NEDD8 conjugation assay, FKA inhibits the formation of NEDD8-Ubc12 conjugates in a dose-dependent manner (Fig. 4D) with an IC_50_ of approximately 5 μM.

### FKA inhibits Cullin1 NEDDylation and induces Skp2 degradation in a wide variety of cell lines regardless of their genetic backgrounds

We also have examined the effect of FKA on Skp2 expression and Cullin1 NEDDylation in different cancer cell lines, including prostate, breast, renal, liver, lung, colon and cervical cancers, melanoma and osteosarcoma. Regardless of their genetic background, FKA treatment decreased the protein levels of Skp2 and Cullin1 NEDDylation in all tested cell lines (Supplementary Fig. 4). These results indicated that FKA is a potent cell active NEDDylation inhibitor leading to Skp2 degradation.

### Dietary feeding of FKA inhibits prostate tumorigenesis in the TRAMP model

**Figure 5:**
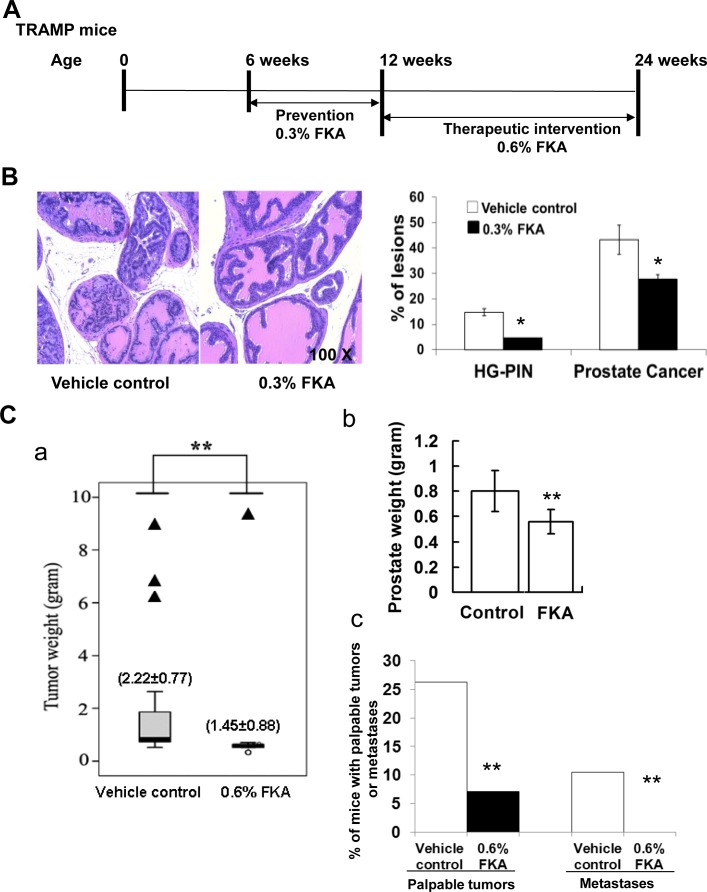
Dietary feeding of FKA inhibits prostate tumorigenesis and blocks metastasis in the TRAMP model **A**. Schematic presentation of prevention and intervention protocols by FKA in the TRAMP model. **B**. Left panel: Representative H&E staining of prostates in 12-weeks-old, vehicle control *versus* 0.3% FKA diet fed TRAMP mice. 100 × magnifications of images are shown. Right panel: Percentages of HG-PIN or adenocarcinoma lesions in the prostates of 12-week-old, vehicle control *versus* 0.3% FKA diet fed TRAMP mice. The prostates were from 8 mice in each group, and the total number of prostate acinus was counted 841 for control group, and 857 for FKA treatment group. Fisher extract tests show Ps<0.05. **C**. Panel a: The genitourinary weights between 24-weeks-old, control (n=25) and 0.6% FKA (n=21) treated TRAMP mice in the intervention protocol are shown; Panel b: The prostate weights of mice from vehicle control (n=22) and 0.6% FKA (n=20) diet fed groups after mice with neuroendocrine carcinomas were excluded; Panel c: Incidences of palpable tumor and visible lung metastasis between 24-weeks-old, vehicle control *versus* 0.6% FKA diet fed TRAMP mice.

To examine whether FKA inhibits different stages of prostate cancer development, a cohort of mice were treated under two different protocols: 1) a prevention protocol in which mice were fed with food supplemented with vehicle control or 0.3% FKA (3g/kg food) starting from 6 weeks old of age and ending at 12 weeks of age; and 2) an intervention protocol in which mice were fed with food that was supplemented with vehicle control or 0.6% FKA starting from 12 weeks of age (when overt cancers are established) and ending at 24 weeks of age (Fig. 5A). Fig. 5B shows that dietary feeding of 0.3% FKA inhibited high-grade prostatic intra-epithelial neoplasia lesions (HG-PIN) by 69% and prostate adenocarcinomas by 43% (Ps<0.05, Fisher exact test). Fig. 5C panel a demonstrates that 12-week FKA feeding of TRAMP mice from age of 12 weeks significantly decreased the tumor burden (control: 2.22±0.77g versus FKA: 1.45±0.88g, *P* < 0.05, Mann-Whitney U and Kolmogorov-Smirnov test). Three mice in control group exhibited neuroendocrine carcinoma, whereas one mouse in 0.6% FKA fed mice has neuroendocrine carcinoma. After excluding the mice with poorly differentiated neuroendocrine carcinomas from the analysis [27], the mean prostate weight for TRAMP mice in the control group is 0.80±0.16 gram *versus* 0.56±0.10 gram in 0.6% FKA treatment group (*p* < 0.01, Fig. 5C panel b). The number of palpable tumors was also decreased by about 73% in TRAMP mice fed with 0.6% FKA food compared to those fed with vehicle control (Fig. 5C, panel c). Approximately 10.5 % (2 out of 19) mice in control group have visible metastases, whereas no metastasis was seen in 0.6% FKA fed mice (*p* < 0.01, fisher exact test). (Fig. 5C). FKA feeding did not result in a decrease in body weight or the weight of major organs. No other signs of toxicity were identified with FKA treatments (Data not shown).

### FKA feeding decreases proliferation, increases apoptosis and affects the expression of Skp2, p27 and NEDD8 in prostate tissues

**Figure 6:**
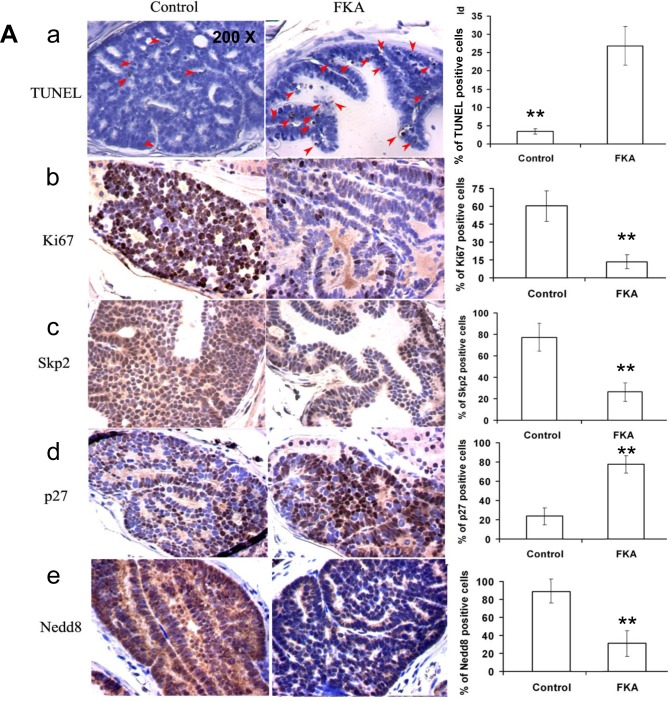

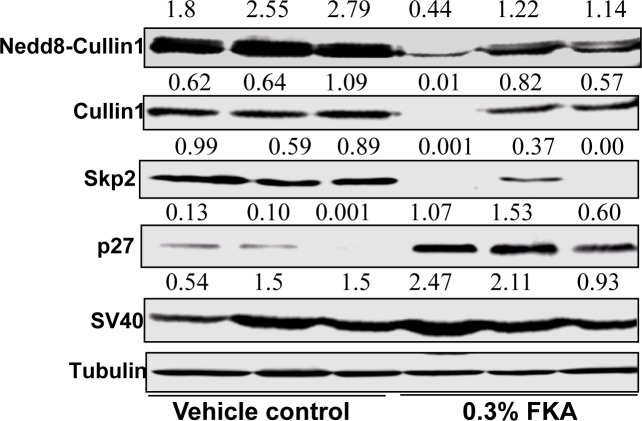
Dietary feeding of FKA induces apoptosis, inhibits cell proliferation and reduces Cullin1 NEDDylation and expression of Skp2 and p27 expression *in vivo* in the prostate of TRAMP mice **A**. A representative immunohistochemistry staining for TUNEL, Ki67, Skp2, P27 and NEDD8 in the prostates of 24-weeks-old, vehicle control *versus* 0.6% FKA diet fed TRAMP mice were shown. 200 x magnifications of images are presented. For TUNEL assay, 20 fields from ten mouse prostates were counted in each group, and the average number of positive stained cells per field is shown. For other staining, 12 fields were counted from eight mouse prostates in each group, and the percentage of positive stained cells was shown. Error bars were standard deviations. **B**. Mouse dorsolateral prostates (n=3 in vehicle control and 0.3% FKA diet groups, none of the samples has neuroendocrine carcinomas) were homogenized in RIPA buffer, indicated protein expressions were examined by Western blot analysis. Tubulin was used as a loading control and SV40 expression shows no difference in transgene expression between control and FKA fed groups.

Microscopic examination of immunohistochemistry-stained prostate tissue sections revealed that the number of apoptotic cells in FKA fed TRAMP mice was 7 folds higher than it is in the control food fed mice (the number of apoptotic cells/field in control versus 0.3% FKA group is 26.9 ± 19.2 versus 3.4 ± 2.5; *P* < 0.01) (Fig. 6A, panel a). The number of Ki67-positive cells in the prostate tissues of FKA-fed TRAMP mice was 60.3% ± 12.7% compared with 13.5% ± 5.8% in those of vehicle control–fed mice (*P* < 0.01; Fig. 6A, panel 6b). This finding suggests both *in vivo* anti-proliferative and apoptotic effects of FKA on prostate tumor tissues, thus slowing the progression of prostate cancer in the TRAMP model.

Consistent with the *in vitro* results described above and the *in vivo* anti-proliferative and apoptotic effect of FKA, immunohistochemistry-stained prostate tissue sections from FKA-fed TRAMP mice also exhibits a significant decrease in both the intensity and the number of Skp2- and NEDD8-positive cells and an increase in p27-positive cells compared with those of vehicle control–fed mice (Fig. 6A, panel c–e).

Western blotting further confirms that that FKA feeding decreases the protein levels of NEDDylated Cullin1, Cullin1 and Skp2 and increases the protein expression of the levels of p27 in prostate tissues of the TRAMP mice (Fig. 6B, upper panels). FKA feeding did not affect the expression of the transgenes SV40T protein and the loading control tubulin (Fig. 6B, lower panels).

## DISCUSSION

Asian/Pacific men who consume a low fat and plant-based diet have the lowest rates of clinical prostate cancer in the world [28, 29]. However, when Asian men migrate to the US, rates of clinical prostate cancer increase [28, 29]. These observations implicate an important role of both environmental factors and dietary habits (such as consumption of low-fat and plant-based diet) in the incidence of prostate cancer. Therefore, one of the strategies for prevention and treatment of prostate cancer has focused on the use of natural and synthetic bioactive food components. Chalcones belong to a specific class of flavonoids, many of which occur in fruits, vegetables and beverages (tea, coffee, beer, wine and fruit drinks) [30]. In this study we have demonstrated that FKA, a predominant chalcone in kava drink, selectively inhibits the growth of pRb deficient cell lines through targeting oncogenic protein Skp2 degradation. In addition, dietary feeding of FKA effectively reduced the occurrence of HG-PIN and prostate adenocarcinomas and blocked tumor metastasis in the TRAMP model.

Skp2 is overexpressed in 86.4 % (64 of 74 samples) of pre-malignant HG-PIN and in 557 of 622 (89.2%) of primary prostate cancer specimens [7]. Skp2 interacts with pRb, Pten, androgen receptor (AR), and H-Ras in prostate cancer [4, 10-12, 31-34], and serves as a critical converging point for these genes in regulation of senescence [10-13]. Skp2 is a pRb target gene and pRb represses Skp2 mRNA expression [33]. Rb also directly binds to the N terminus of Skp2 to interfere with its function [33]. Nuclear Pten enhances APC/Cdh1 activity that degrades Skp2 [32]. Therefore, the elevated Skp2 levels duo to the pRb or Pten loss were suggested to be a main contributor to tumorigenesis and cancer progression. Indeed, genetic inactivation of Skp2 triggered a potent senescence response to a restriction of tumorigenesis to the prostate in the Pten deficient mice [13], and abolished spontaneous tumorigenesis in Rb1 (+/−) mice [10-12]. Since allelic loss or reduced expression of Rb in 25% to 50% of human prostate cancer [14, 35], and Pten deletion and/or mutations in 30% of primary prostate cancer and 63% of metastatic prostate cancer [15] were associated with more frequent clinical recurrence, Skp2 may serve an “Achilles' heel” type of drug target to effectively prevent or treat pRb and/or Pten deficient prostate cancer. Here, we have shown that FKA effectively down-regulated Skp2 protein levels with corresponding accumulation of p27/Kip1 in all tested cancer cell lines regardless their genetic background (Fig. 2A and Supplementary Fig. 2) and *in vivo* in the prostate of the TRAMP mice (Fig. 5). This result suggests that Skp2 is a main target for the anti-cancer mechanisms of FKA. FKA deserves further investigations for targeting prevention or intervention in subgroups of prostate cancer high-risk populations or patients with pRb or Pten deficiency.

The down-regulation of Skp2 by FKA is not through transcriptional regulation, but by accelerating its degradation. Since proteasome inhibitor MG132 was reported to regulate Skp2 expression at the transcription level [36], we transfected PC3 cells with Myc-tagged Skp2, which is driven by an exogenous CMV promoter. Therefore, the protein level of Myc-Skp2 was not affected by MG132 treatment alone, but MG132 completely reversed the FKA-induced inhibition of Myc-Skp2 expression (Fig. 2B). This result suggests that FKA induced Skp2 degradation is dependent on proteasome function. We have also shown that FKA induces Skp2 ubiquitination. It is likely that FKA degrades Skp2 through a proteasome-dependent and ubiquitin-mediated pathway.

Skp2 ubiquitination and degradation has been reported either through a Cullin1-dependent autocatalytic mechanism or by the Cdh1/APC/C complex [23, 24]. Our results did not support the involvement of the Cdh1/APC/C complex in the FKA mediated Skp2 degradation, as FKA itself decreased the expression of Cdh1 and knockdown of Cdh1 expression by siRNA did not prevent the FKA mediated down-regulation of Skp2 expression (Fig. 3A and 3B). In addition, pRb has recently been shown to be required for the Cdh1/APC/C complex mediated Skp2 degradation [37]. However, FKA down-regulates the expression of Skp2 regardless the genetic background of tested cell lines in PC3 and 22Rv1 cells expressing wild-type Rb and DU145 cells harboring Rb deletion (Fig. 2A and supplementary Figure 2). This further supports that other Rb and Cdh1 independent mechanisms are involved in FKA-mediated Skp2 degradation.

The F-box domain of Skp2 is required for the auto-ubiquitination of Skp2 by the Skp1- Cullin1-Roc1 complex [24]. When the F-box domain was deleted or Cullin1 was knocked out, Skp2 expression remained higher under serum starvation condition and could not be ubiquitinated *in vitro* [24]. We have shown that FKA was not able to down-regulate the expression of F-box deleted Skp2 and that ectopic expression of B-box deleted Cullin-1 restored the FKA-mediated down-regulation of Skp2 expression (Fig. 3C and 3D). The autocatalytic ubiquintination and degradation of Skp2 also requires the ability of the Cullin1 to recruit Cdc34 and Roc1/Rbx through its B-box motifs [24]. These results support that functional Cullin1 is required for the FKA mediated Skp2 degradation.

The activity of the Cullin1-containing ubiquitin protein ligase complex SCFSkp2 for p27Kip1 ubiquitylation is stimulated by linking the ubiquitin-like protein NEDD8 to Cullin1. Similar to ubiquitination, NEDDylation of Cullin1 is involved in sequential attachment of NEDD8 to a thiolester within a specific cysteine site of NEDD8-E1 (NEDD8-activating enzyme, NAE, APP-BP1/Uba3) and NEDD8-E2 (Ubc12) [25]. Recently, the NEDD8 pathway has become an important target for cancer therapy. MLN4924 is a first-in-class inhibitor of the NEDD8 pathway by binding to and inhibiting NAE [25]. Based on its promising preclinical efficacy, MLN4924 has been advanced to Phase I clinical trials for treatment of multiple human malignancies [38]. We have demonstrated that FKA docks into the ATP binding pocket of the APPBP1–Uba3–NEDD8 complex and inhibits Ubc12 NEDDylation in *in vitro* system and NEDDylation of Cullin1and Ubc12 in all tested cell lines, as well as in the prostate tissues of the TRAMP mice. Further studies are in progress to determine whether FKA directly bind to Uba3 or Ubc12 and interferes with the process of NEDDylation. In addition, it is worth to mention: The results from analysis of *Uba3^−/−^* or *Skp2^−/−^* embryos revealed that both the NEDD8 system and Skp2 are critical for the control of the mitotic and endoreduplicative cell cycle progression [39]. These results suggested the NEDD8 system and Skp2 may form a yet to be defined signaling axis in regulation of the control of the mitotic and endoreduplicative cell cycle progression. Therefore, further studies are in progress to determine whether there is a potential direct interaction between the NEDDylation pathway and Skp2 degradation. Since Skp2 overexpression is frequently observed in many cancers and associated with poor prognosis [2, 4], as well as play a critical role in tumorigenesis, cell cycle regulation, metastasis and metabolism [10-15], further elucidation in the novel mechanism of Skp2 degradation that are independent of the Cdh1/APC/C complex may open a new avenue for designing cancer-specific prevention and therapy.

The transgene SV40T in the TRAMP model can bind to and inactivate pRb and p53 tumor suppressor proteins leading to cell transformation [40]. Skp2 is strongly expressed in the hyperplastic/dysplastic and prostate carcinoma regions in the TRAMP model in our study. This model is therefore suitable for studying agents that inhibit pRb deficient prostate cancer through targeting Skp2. We have shown that dietary FKA effectively inhibits all stages of prostate cancer development, which reduce the incidences of both HG-PIN and prostate carcinoma. Importantly, the mechanism of prostate cancer inhibition by dietary FKA is not through down-regulation of the transgene as the protein levels of SV40T are equal between vehicle control and FKA fed mice. In addition, dietary FKA completely blocked distant organ metastasis. However, the incidence of visible distant organ metastasis is relatively low (about 10.5%) in the TRAMP mice generated in our study. Our study of dietary FKA on tumor metastasis in the TRMAP model may be limited by not enough sample size for studying metastasis. Therefore, this result requires further validation with a primary endpoint of metastasis using a larger sample size. In our recent studies, we have also demonstrated that dietary FKA increased the survival of bladder tumor bearing mice and reduced tumor growth in the UPII-SV40 bladder specific transgenic model. In all of our studies, dietary FAK did not cause any body weight loss or changes in food and water consumption and normal organ functions [20, 41]. Further pharmacokinetics and tissue distribution studies found that FKA is concentrated in urine and accumulates at higher concentrations in liver, kidney and prostate than in lung via dietary supplementation [20, 42]. Therefore, compared to other methods (i.e. intraperitoneal injection and gavage) of FKA delivery, dietary FKA is effective in inhibiting tumorigenesis in both bladder and prostate cancer transgenic carcinogenesis model with satisfactory safety profile.

In the present study, dietary FKA was found to be effective in inhibition of proliferation and in marked induction of apoptosis in pre-cancerous and cancerous cells in the TRAMP model. This result is similar with our recent observations in the FKA fed bladder cancer model [20]. Also in consistency with the *in vitro* mechanisms of FKA's action, dietary FKA results in down-regulation of Skp2 and NEEDylation of Cullin1 and up-regulation of p27/Kip1 in the prostate tissues of the TRAMP model.

**Figure 7:**
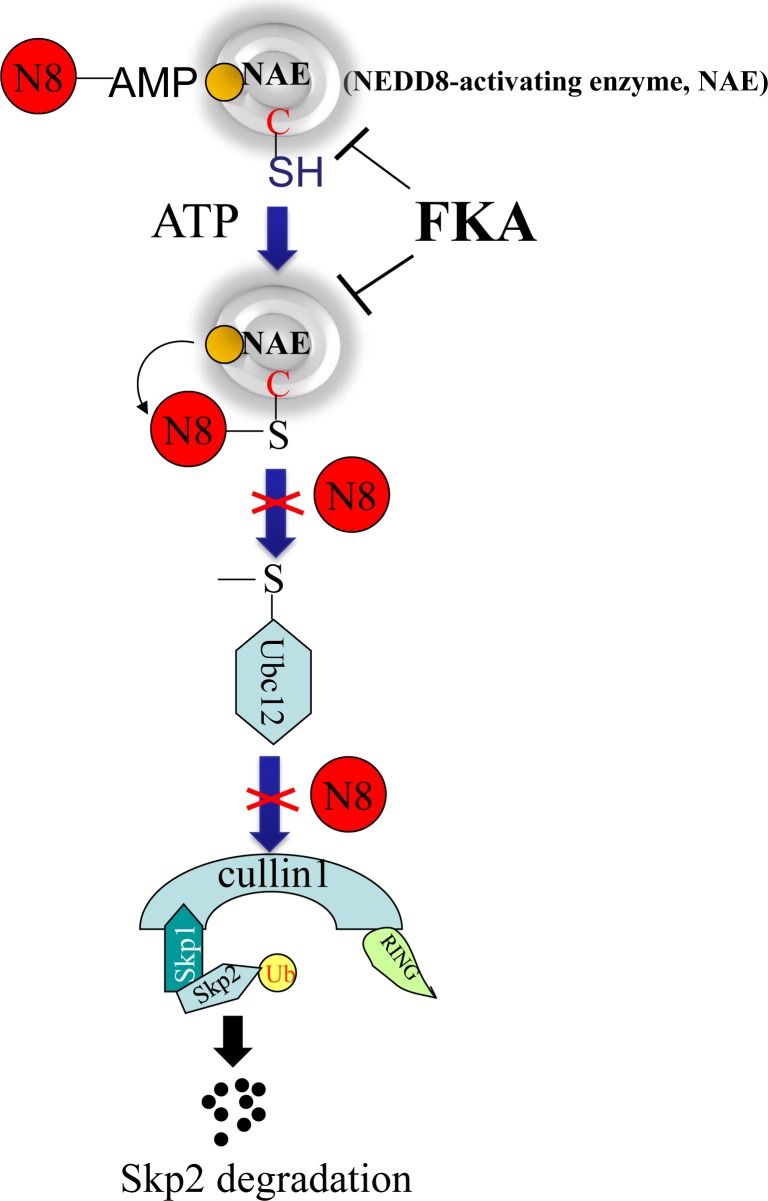
Model of a mechanism by which FKA binds to the NAE complex and inhibits the transfer of NEDD8 from NAE to Ubc12 and Cullin1 The deNEDDylation of Cullin1by FKA then results in Skp2 auto-ubiquitination and degradation.

In summary, FKA selectively inhibits the growth of pRb deficient cells through inducing deNEDDylation and Skp2 degradation. The FKA induced Skp2 degradation or down-regulation is independent of Cdh1 and the genetic backgrounds of tested cells lines, such as pRb deletion or p53 mutations, but requires a functional cullin1. Our data are consistent with the model shown in Figure 7. FKA docks into the active site of the NAE complex and inhibits the transfer of NEDD8 to Ubc12 and Cullin1. Then, Cullin1 deNEDDylation results in Skp2 auto-ubiquitination and degradation. Further investigation of this novel Cullin1-mediated Skp2 degradation pathway may present new opportunity for targeting Skp2 in prevention and treatment of cancer. The observed inhibition of prostate tumorigenesis and subsequent metastasis by dietary FKA is caused by selective apoptotic death and anti-proliferative effects. The potential use of FKA with an excellent safety profile is a major plus in the planning of translational development of FKA-based prevention and therapy.

## MATERIALS AND METHODS

### Cell lines, compounds and reagents

All the prostate cancer cell lines and other type cancer cell lines were obtained from American Type Culture Collection (ATCC) (Manassas, VA); except C4-2B cell line was from Urocor Inc. (Oklahoma City, OK). These cells were cultured in basic medium with 10% fetal bovine serum (FBS) as described in our previous publication [43]. *Rb^+/+^* and *Rb^−/−^* MEFs were obtained from Dr. Wen-Hwa Lee at the University of California, Irvine and cultured in high-glucose Dulbecco's modified Eagle medium with 10% FBS [44]. *Rb^+/+^* and *Rb^−/−^* MPECs were from Dr. Scott D. Cramer at Wake Forest University School of Medicine, and were cultured in Dulbecco's modified Eagle's medium(DMEM)/F12 supplemented with 1% FBS [45]. All cell lines used in this study were within 20 passages after receipt. The cell lines were tested and authenticated by ATCC or Urocor Inc. All cells lines were also tested for known species of mycoplasma contamination using a kit from LONZA Inc. (Walkersville, MD). FKA (99%) were isolated from kava extracts by LKT Laboratories, Inc. (St. Paul, MN). Antibodies against Ubc12, Skp1, SV40 and tubulin were from Santa Cruz Biotechnology, Inc. (Santa Cruz, CA). Skp2, Flag tag and HA tag antibodies were purchased from Invitrogen (Grand Island, NY). Myc tag and V5 tag antibodies were from Cell Signaling (Boston, MA). Cdh1, Cullin-1 and NEDD8 antibodies were from Abcam (Cambridge, MA). p27/Kip1 antibodies was from BD Biosciences (Billerica, MA). 3-(4, 5-dimethylthiazol-2-yl)-2, 5-diphenyltetrazolium bromide (MTT) was from Sigma. RNAzol B was purchased from Tel-Test (Friendswood, TX.). The Reverse Transcription System kit and was from Promega (Mandison, WI). A quantitative RT-PCR kit was from Bio-Rad (Hercules, CA).

### MTT assay [22]

Cells were plated at a density of 2 × 10^4^ per well in 24-well culture plates for 24 hours, and then treated as indicated in the figures. After treatments, 1 mg/mL MTT in 20% PBS and 80% culture medium (v/v) was added to each well for 2 hours, and the absorbance was determined at 570 nm. Dose–response curves for growth inhibition were generated as a percentage of vehicle-treated controls.

### Western blot analysis [22]

Clarified protein lysates (20-100 μg) were denatured and resolved by 8-16% SDS-PAGE. For non-denatured western blot, lystate were mixed with non-reducing loading buffer and resolved in non-denaturing gel (Biorad, CA). Proteins were transferred to nitrocellulose membranes, and probed with indicated antibodies and visualized by an enhanced chemiluminescence detection system. The densities of Western blotting bands were semi-quantified by Quantity-One software (Biorad), and were adjusted for loading controls β-actin or tubulin.

### Plasmid and siRNA transfection

Plasmid of pcDNA-Skp2/myc was from Addgene No. 19947 [46], and was transfected with Fugene 6 from Roche (Indianapolis, IN), and stable clones were screened and used in the experiments. Delta-B box Cullin-1 (Dominant negative Cullin-1 (1-456) plasmid was a kindly gift from Dr. Zhen-Qiang Pan (Derald H. Ruttenberg Cancer Center, New York) [47]; delet-F-box-Skp2-V5 was a kindly gift from Dr. Thilo Hagen (National University of Singapore) [48]. All the siRNAs, including siRb (GS5925) and siCdh1 (GS51343), were from Qiagene (Valencia, CA). All the transient transfections were performed using Lipofectamin 2000 from Invitrogen.

### TRAMP mice study

Animal care and treatments were in accordance with institutional guidelines and the approved protocol by University of California, Irvine (protocol #:2007-2740).

Female hemizygous C57BL/TGN TRAMP mice and male C57BL/6 mice were purchased from The Jackson Laboratory. Mice were genotyped by a PCR method, and male hemizygous mice were used in the study. FKA was commercially formulated into AIN-93M rodent food (Dyets, Inc.). In the prevention protocol, TRAMP mice were fed with 0.3% (3 g /kg food) FKA containing food, starting at age of 6 weeks old and ending at age of 12 weeks old. In the intervention protocol, 12 weeks old mice were fed with 0.6% FKA containing food until they died or reached at age of 24 weeks old. TRAMP mice in vehicle control groups were fed with AIN-93M rodent food.

The mice and food were weighted weekly. At the end of treatment, all mice were euthanized by CO_2_ asphyxiation. Serum samples were obtained by cardiac puncture. All major organs will be inspected for frank toxicity and weighed. Systematic necropsy for gross and microscopic examination was carried out. Necropsy was documented with quantitative and qualitative descriptions of the prostate, liver, lymph nodes, and tissues showing any visible abnormality. All organs will be removed and fixed in formalin for standard H& E slide preparation and examination [20]. Prostates were dissected into coagulating gland, seminal vesicle, lateral, dorsal, and ventral prostate and fixed with 10% buffered formalin. The sections were cut from paraffin-embedded tissues and stained with H&E to classify the PIN lesions (PIN I–IV) classification according to Dr. Cardiff's description [49].

### *In vitro* NEDD8 conjugation assay [25]

The *in vitro* NEDD8 conjugation assay kit was purchased from BostonBiochem (Cambridge, MA). A master mix of 0.4 μM APPBP1/Uba3 (NEDD8 ligase E1), 12.5 μM UbcH12 (NEDD8 ligase E2) and 62.5 μM NEDD8 were prepared in the reaction buffer (pH8.0, 50mM Hepes and 50 mM NaCl in final reaction) and distributed to individual tubes with a volume of 15 μl. A series of dilutions of FKA were made in DMSO. One microliter of FKA or DMSO were added to the indicated tubes and mixed well. The reactions were started by adding 2.5 mM Mg^2+^ and 1 mM ATP (4 μl in mixture), except the negative control tube was added by equal volume of ddH_2_O. The reaction tubes were incubated in 37°C for 30 minutes and stopped by adding 5 μl 25 mM EDTA. Non-reducing western blot was performed with anti-UbcH12 antibody to detect both Ubc12 bands and Nedd8 conjugated Ubc12 bands.

### Molecular docking

The X-ray crystal structure of the quaternary amyloid precursor protein-binding protein (APPBP1) – Ubiquitin-Like Modifier Activating Enzyme 3 (UBA3)–NEDD8–ATP complex was retrieved from the Protein Data Bank (PDB code: 1R4N) [26] and processed in order to remove the ligands/water molecules with the module of Biopolymer (Sybyl-x2.0 program) [50]. Hydrogen atoms were added to the protein structure using standard geometries with Biopolymer module. Pre-Dock minimization was performed to minimize contact between hydrogen atoms, keeping the heavy atoms fixed at their crystallographic positions.

The docked ligand was built using the Builder module of Sybyl-x2.0 and the geometry of the small molecule was optimized using Tripos force field and Gasteiger-Huckel charge, with the convergence criteria 0.05 kcal/(mol*A). Then the ligand was manually docked into the putative ATP binding sites using the flexible Surflex-Dock methodology with considering the lowest 10 conformers in energy. Post-Dock minimization was used to minimize the resulting ligand–APPBP1–UBA3–NEDD8 complexes. The purpose of Surflex-Dock is to search for favorable binding configurations between a small, flexible ligand and a rigid macromolecular target. All the calculations performed in Mac workstation.

### Immunohistochemistry [20, 43]

Terminal deoxynucleotidyl transferase dUTP nick end labeling (TUNEL) assay was conducted with the kit from Promega (Madison, WI). For other antigens' staining, antigen was retrieved using 10 mM sodium citrate buffer, pH 6.0, containing 0.05% (w/v) Tween-20, in pressured cook at 121°C for 1 min and stained with anti-mouse Ki67 (Abcam, 1:200), Skp2 (Abcam, 1:200), p27 (BD bioscience, 1:50), Nedd8 (Abcam, 1:100). Staining was visualized with diaminobenzadine using the Cell and Tissue Staining kit (R&D Systems).

### Statistical analysis

Mean, standard deviation (SD), standard errors (SE) and confidence intervals of all quantitative data were computed in Excel. Organ and body weight comparisons between vehicle control and FKA treatments were accomplished using either analysis of variance (ANOVA) or Student's t test followed by the Bonferroni t test for multiple comparisons. Prostate and genitourinary weights between vehicle control and FKA treatments were compared using Mann-Whitney U and Kolmogorov-Smirnov test. The χ2 or fisher exact test was used to compare the percentages of mice with different pathologic stages or with palpable tumors between vehicle control and FKA treatments. All statistical measures were two-sided, “* ” and “**” stand for P < 0.05 and <0.01 in all figures, respectively.

## SUPPLEMENTARY MATERIAL FIGURES


