# Supplementary material for: Flavokawain A induces deNEDDylation and Skp2 degradation leading to inhibition of tumorigenesis and cancer progression in the TRAMP transgenic mouse model
Source: Oncotarget. 2015 Oct 19;6(39):41809–24. doi: 10.18632/oncotarget.6166 (PMC4747190; doi:10.18632/oncotarget.6166)
Supplement: Supplementary file 1 [file oncotarget-06-41809-s001.pdf]

Flavokawain A induces deNEDDylation and Skp2 degradation leading to inhibition of tumorigenesis and cancer progression in the TRAMP transgenic mouse model

Supplementary Material

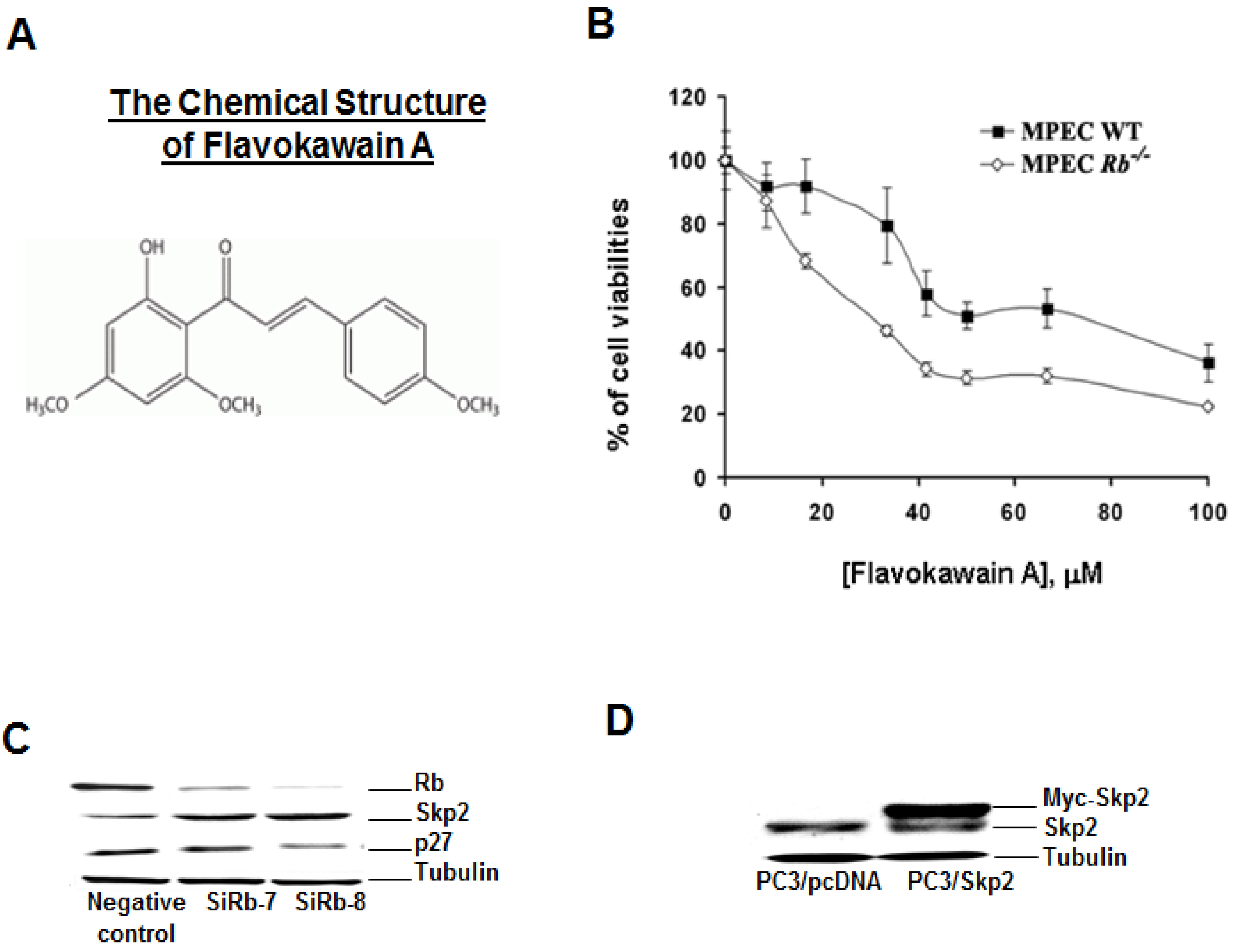

Supplementary Figure 1. (A) The chemical structure of FKA . (B) *FKA preferentially inhibits the growth of Rb knockout vs. wild-type MPECs.* Rb wild-type and Rb knockout MPECs were treated with 0.1% DMSO or indicated concentrations of FKA for 72 hours. Cell viabilities were measured by MTT assays. Points, means of four independent plates; bars, SE. (C) Western blotting analysis of pRb, Skp2 and p27 expression in 22Rv1 cells that were transfected with siRNA control or two different Rb siRNAs. (D) Stable overexpression of Myc tagged Skp2 in PC3/Skp2 vs. PC3/pcDNA cells was shown by Western blotting analysis using anti-Skp2. β-tubulin is a loading control.

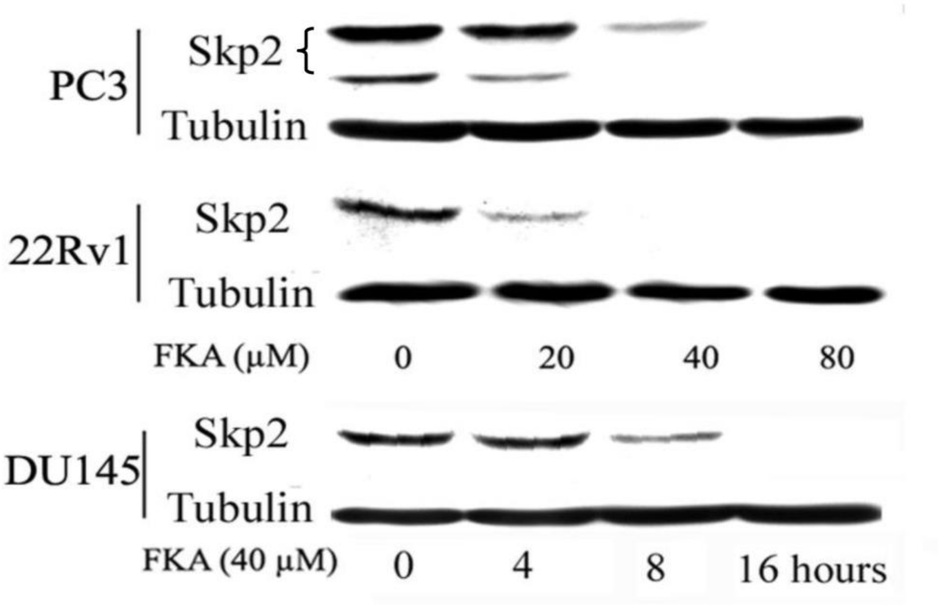

Supplementary Figure 2. PC3 and 22Rv1 cells were treated with 0.1% DMSO or indicated concentrations of FKA for 24 hours, and DU145 cells were treated for different time periods. A representative Western blotting analysis of Skp2 protein expression was shown.

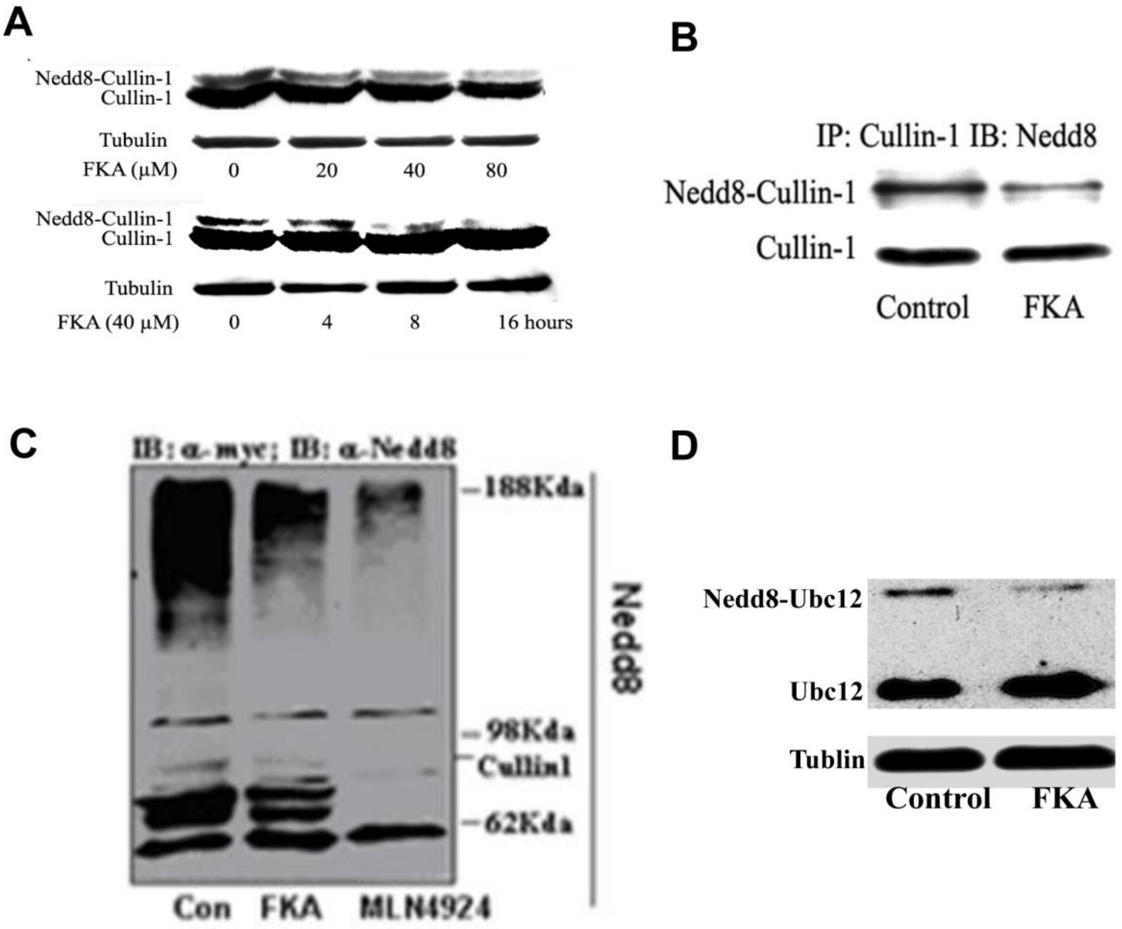

Supplementary Figure 3. A. Western blotting analysis of Cullin-1 neddylation in DU145 cells. DU145 cells were treated with 0.1% DMSO or different concentrations of FKA for 24 hours or 40 mM FKA for different periods of time. B. Immunoprecipitation of Cullin1 and Western blotting analysis of NEDDylation reveals that 40 micromolar FKA treatment of DU145 cells for 24 hours resulted in a reduction of Cullin-1 neddylation. C. DU145 cells were transiently transfected with myc-Cullin1 for 48 hours and then treated with vehicle control (0.1% DMSO), 40 micromolar FKA or 1 micromolar MLN4924 for 24 hours. Immunoprecipitation of myc-Cullin1 by anti-myc tag antibody followed by Western blotting analysis of NEDD8 shows that FKA decreases Cullin-1 NEDDylation. D. Western blotting analysis Ubc12 expression shows a decreased NEDDylation of Ubc12 accompanied with an increase in unNEDDylated Ubc12 in DU145.

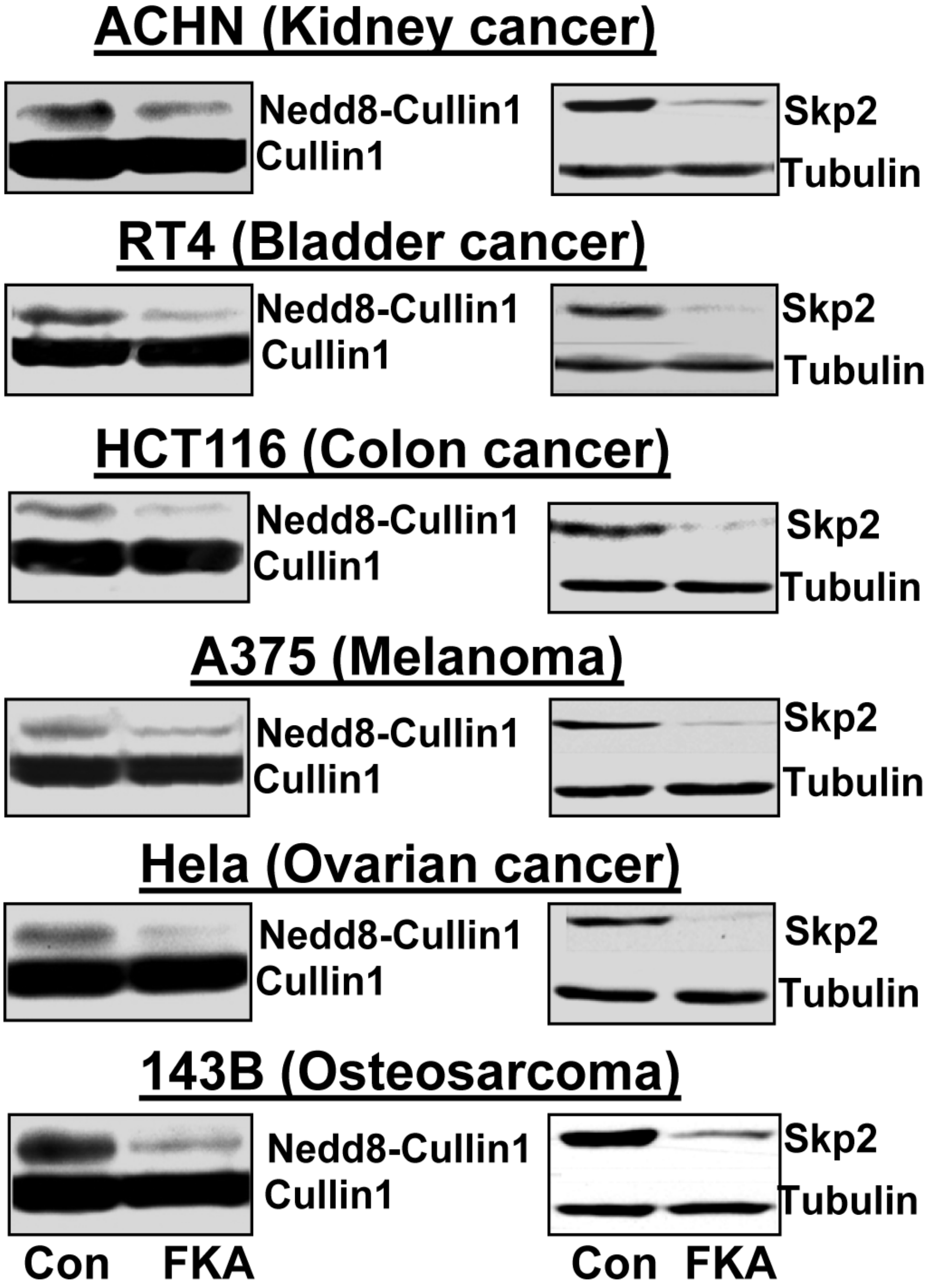

Supplementary Figure 4. FKA inhibits Cullin1 NEDDylation and down-regulates Skp2 expression in all tested cell lines. Western blotting analysis of Cullin1 NEDDylation and Skp2 expression after 40 micromolar FKA treatment for 24 hours.
